# Supplementary material for: Codon Usage and Splicing Jointly Influence mRNA Localization
Source: Cell Syst. 2020 Apr 22;10(4):351–362.e8. doi: 10.1016/j.cels.2020.03.001 (PMC7181179; doi:10.1016/j.cels.2020.03.001)
Supplement: Document S1. Figures S1–S6 and Tables S1 and S2 [file mmc1.pdf]

Cell Systems, Volume *10*

Supplemental Information

Codon Usage and Splicing Jointly Influence mRNA Localization

Christine Mordstein, Rosina Savisaar, Robert S. Young, Jeanne Bazile, Lana Talmane, Juliet Luft, Michael Liss, Martin S. Taylor, Laurence D. Hurst, and Grzegorz Kudla

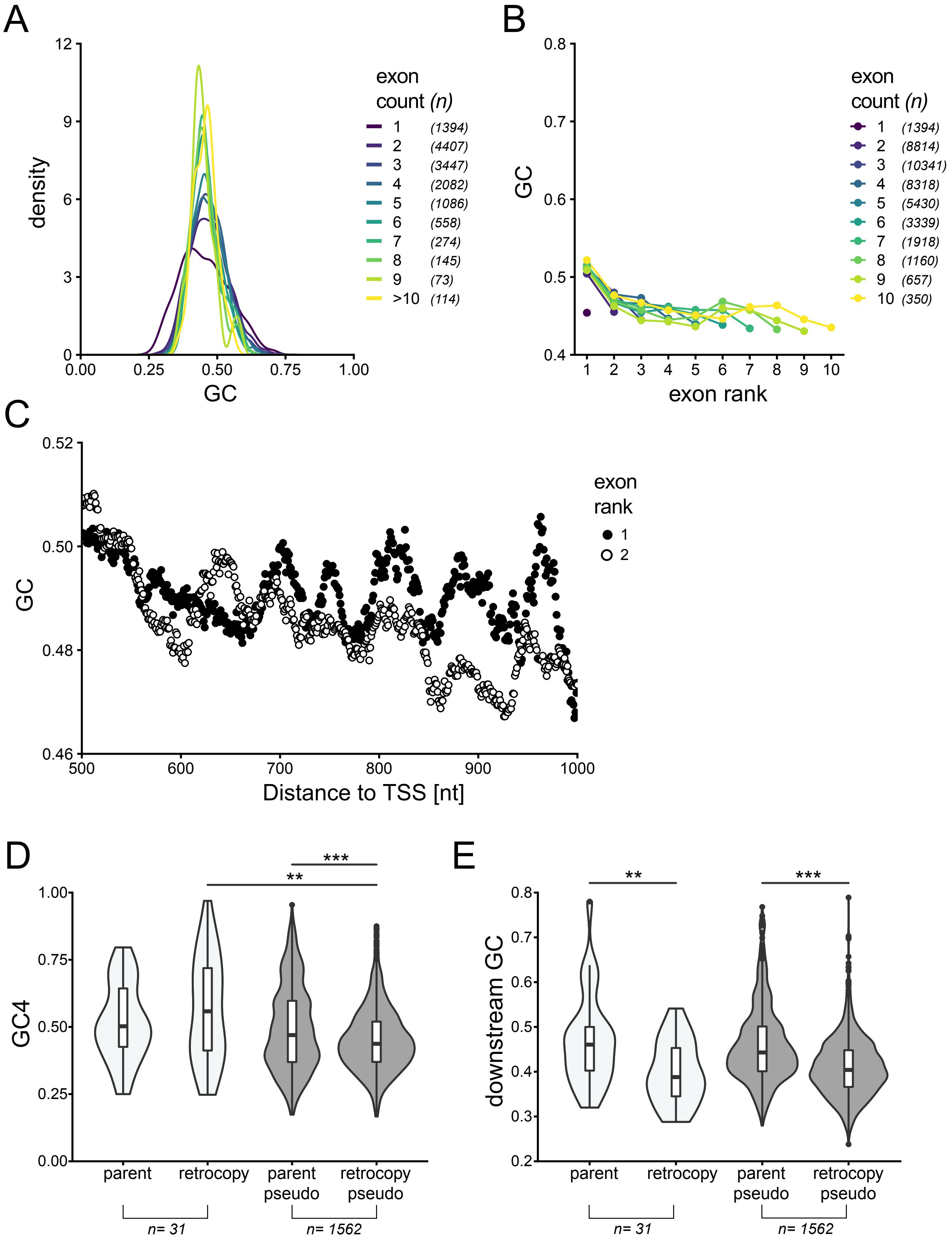

Figure S1. GC variation amongst lncRNAs and parent-retrogene pairs and their downstream sequence, related to Figure 1.

Figure S1 (continued) (A) GC distribution of human long non-coding RNA genes, grouped by number of exons per gene. The Y axis indicates the proportion of genes within a given range of GC, calculated using the ggplot2 geom_density() function.
(B) Mean GC content in non-coding exons, grouped by exon position (rank) and by number of exons per gene. (C) Mean GC within exons of rank 1 (black dots) or rank 2 (white dots) downstream of the transcription start site (TSS). (D) GC4 content distribution across parent and retrogene pairs conserved between human and macaque. White violins indicate pairs for which retrocopies are classed as functional ($p=0.26$, $n=31$, two-tailed Wilcoxon signed-rank test), whereas grey violins correspond to pairs in which the retrocopy is classed as non-functional pseudogene ($p < 2.2\times10^{-16}$, $n=1562$, two-tailed Wilcoxon signed-rank test). For the human-macaque set, the difference in GC4 between parents and functional copies is in the expected direction but not significant. (E) Violin plot showing GC content within a window between 2000 and 3000nt downstream from the stop codons of functional (white, $p=9.3\times10^{-4}$, $n=31$, two-tailed Wilcoxon signed-rank test) and non-functional (grey, $p<2.2\times10^{-16}$, $n=1562$, two-tailed Wilcoxon signed-rank test) parent-retrogene pairs conserved between human and macaque.

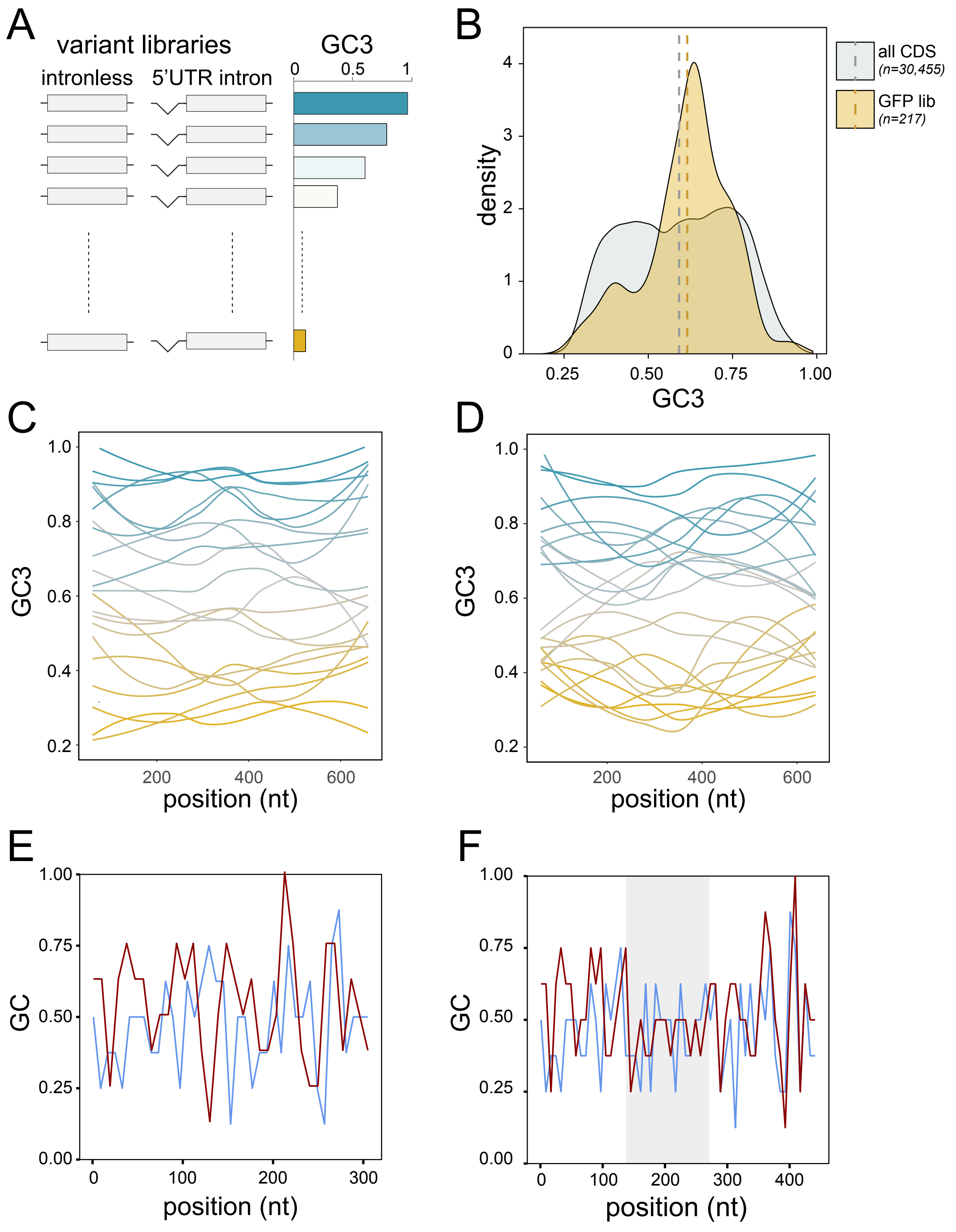

Figure S2. GC content variation amongst endogenous genes and reporter libraries, related to Figure 2. (A) Libraries of reporter genes with random synonymous codon usage were designed to cover a broad range of GC3 content variation. Variants were expressed with and without a synthetic 5' UTR intron. (B) GC3 content distribution amongst human consensus coding sequences (CDS; grey) in comparison to the GFP variant library used in this study (GFP lib; orange). Dashed lines indicate the mean GC3 for each data set. (C-D) Loess-smoothed GC3 profiles along the 22 GFP variants (C) and 23 mKate variants (D) that were analysed by spectrofluorometry (Figure 2). (E) Sliding window analysis of GC content in 5' UTRs of intronless expression cassettes utilised in this study. Blue: pCM3 (transient transfection, no intron); red: pcDNA5/FRT/TO/DEST (stable transfection, no intron). (F) As above, intron-containing expression cassettes. Blue: pCM4 (transient transfection, with intron); red: pcDNA5/FRT/TO/DEST/INT (stable transfection, with intron). Grey shading indicates the position of the synthetic intron.

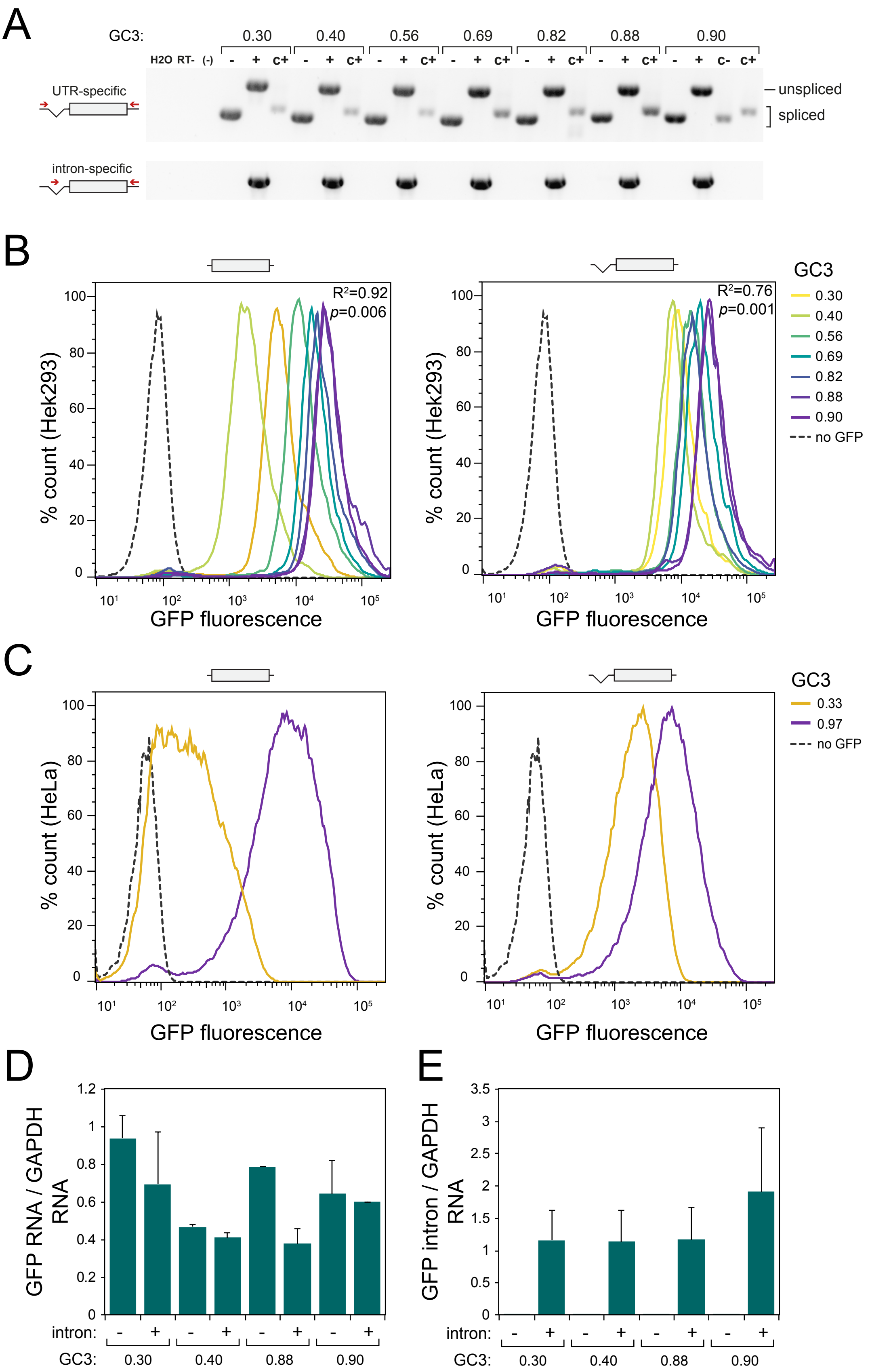

Figure S3. Effect of GC content on expression of fluorescent reporter genes in stably transfected cell lines, related to Figure 2.

Figure S3 (continued). (A) RT-PCR using total RNA from HEK293 Flp-In cell lines stably expressing several variants of GFP with a broad GC3 range (GC3 range: 0.3 – 0.9) and containing the same 5' UTR intron as used throughout this study. PCR was performed using either UTR-specific primers that detect spliced as well as unspliced GFP transcripts (upper gel, labelled 'UTR-specific)), or primers that exclusively detect unspliced transcripts (lower gel, labelled 'intron-specific'). Plasmids containing the respective GFP expression cassettes, both with or without UTR intron, are shown as controls. (B-C) Flow cytometry measurements of GFP variants covering a broad range of GC3 variation in stably transfected HEK293 Flp-in (B) and HeLa Flp-in (C). (D-E) qRT-PCR measurements of nascent RNA isolated using 4sU labelling from 2 GC-poor (GC3=0.3 and 0.4) and 2 GC-rich (GC3=0.88 and 0.9) GFP variants, expressed as unspliced or spliced constructs. GFP RNA levels were measured using 3' UTR specific primers (D, full length transcripts) and intronic RNA levels (E, pre-mRNA). Data points represent the mean of 2 independent experiments, -/+ SD.

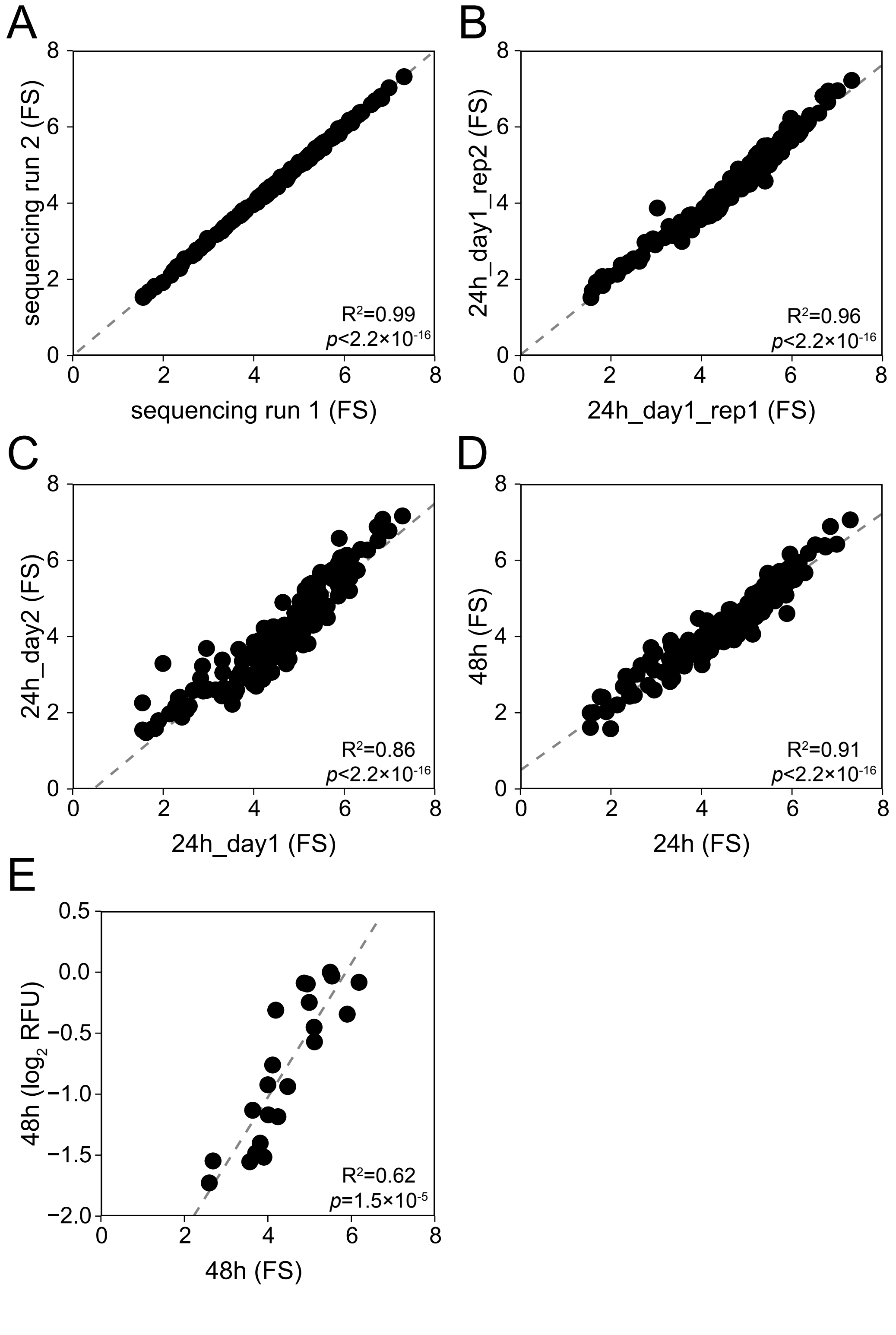

Figure S4. Reproducibility of Flow-seq experiments in HeLa cells (unspliced GFP variants), related to Figure 3.

(A-E) GFP Flow-Seq fluorescence scores (FS), calculated as described in the Methods section. (A) Re-sequencing of the same amplicon-library. (B-C) Replicate Flow-seq experiments performed on the same day (B) or different days (C). (D) Flow-Seq experiments performed on the same pool of cells, 24h and 48h after the induction of GFP expression. (E) Correlation between fluorescence measurements of 22 GFP variants obtained in the HeLa GFP pool cell line by Flow-Seq (X axis) and in transiently transfected HeLa cells by spectrofluorometry (Y axis, data from Figure 2).

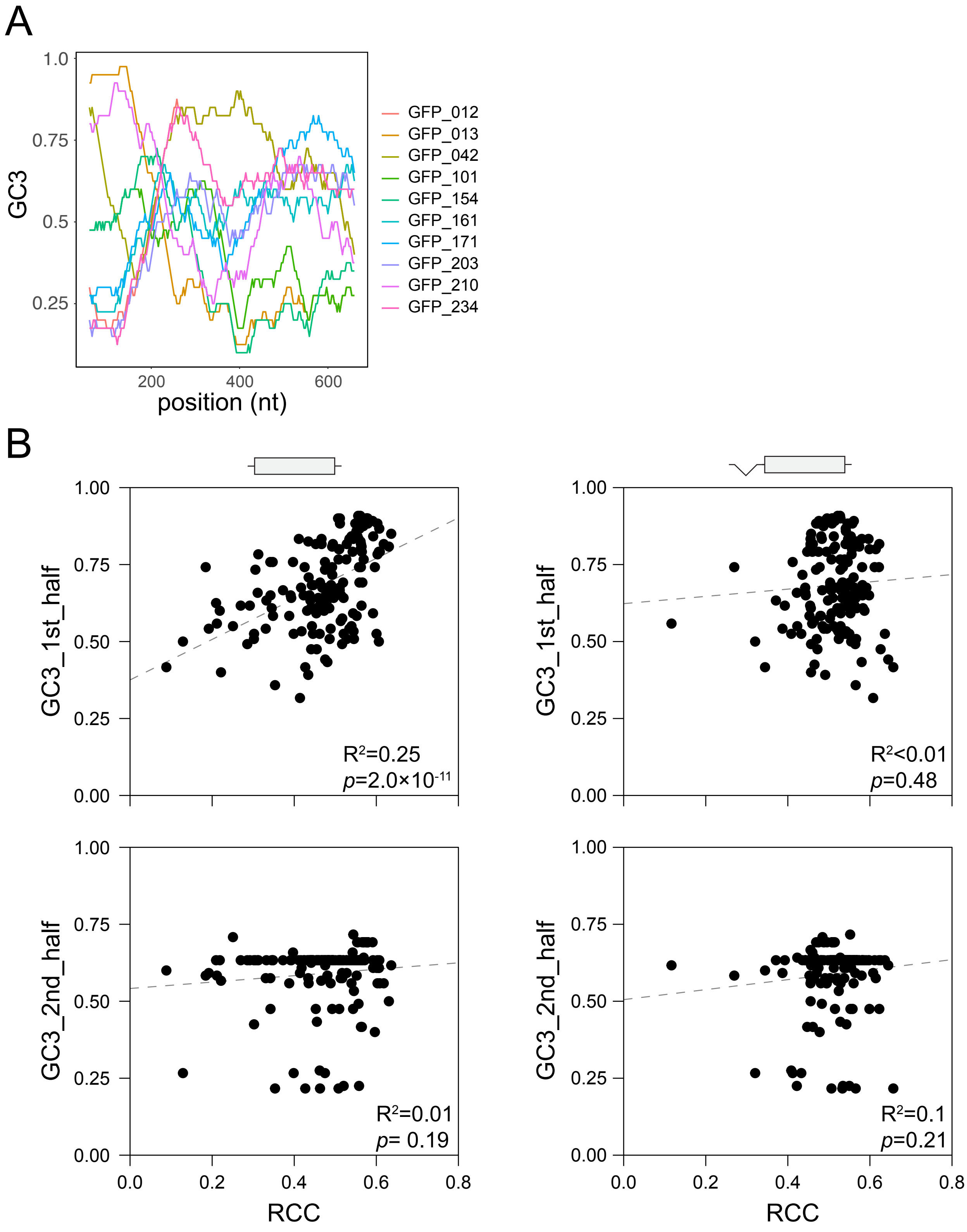

Figure S5. Position-specific effects of GC content on expression, related to Figures 3 and 4. (A) Sliding window analysis of GC3 content in selected GFP variants used in the pooled amplicon sequencing experiments. (B) Correlations between the GC3 content in the 1st (nt 1-360) and 2nd (nt 361-720) halves of GFP variants and their relative cytoplasmic mRNA concentrations (RCC).

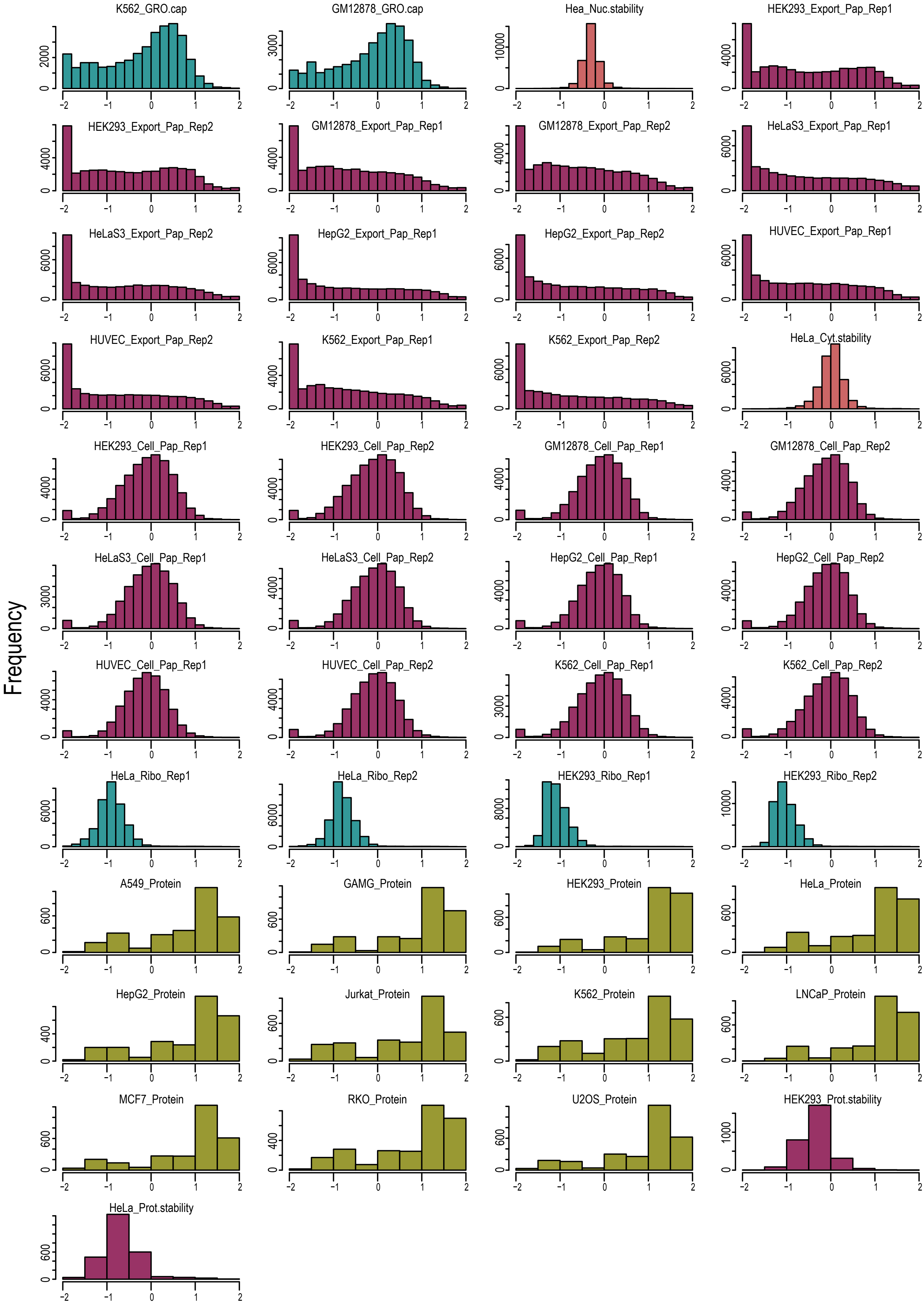

Figure S6. Distribution of RNA and protein expression data used in regression modelling, related to Figure 6.

Figure S6 (continued) Human RNA and protein expression data were extracted from various databases, filtered and normalized as described in Table S1 and STAR Methods. The histograms show the distributions of preprocessed expression measurements.

Table S1. Sources of human gene expression data, related to Figure 6. The cellular process to be quantified is indicated above the table, and the experimental techniques and data sources are indicated below. Each dot indicates an experimental replicate measurement.

	Transcription	nuclear stability	cytoplasmic stability	RNA levels	RNA export	Translation	Protein levels	Protein stability
K562	●			●●	●●		●	
Gm12878	●			●●	●●			
HeLa		●	●	●●	●●	●●	●	●
Hek293				●●	●●	●●	●	●
Huvec				●●	●●			
HepG2				●●	●●		●	
A549							●	
GAMG							●	
Jurkat							●	
LnCap							●	
MCF7							●	
RKO							●	
U2OS							●	
data type	GRO-cap	CAGE-seq: Mtr4 KD/ EGFP KD	CAGE-seq: Rrp40 KD/ Mtr4 KD	RNA-seq	RNA-seq	Ribo-seq	Mass-spec	Mass-spec/Ribo-seq
data source	ENCODE	Andersson et al., 2014	Andersson et al., 2014	Hek293: this study; all others: ENCODE	Hek293: this study; all others: ENCODE	ENCODE	Geiger et al., 2012	Geiger et al., 2012; ENCODE

Table S2. List of primer sequences, related to STAR methods.

MiSeq library + sequencing	5' → 3'
PE_PCR_left	AATGATACGGCGACCACCGAGATCTACACGCTGGCACGCGTAAGAAGGAGATATAACCATG
S_index1_right_PEPCR	CAAGCAGAAGACGGCATACGAGATCGTGATGTGACTGGAGTTCAGACGTGTGCTCTTCCGATCTATGTGCAGGGCCGCGAATTC
S_index2_right_PEPCR	CAAGCAGAAGACGGCATACGAGATACATCGGTGACTGGAGTTCAGACGTGTGCTCTTCCGATCTATGTGCAGGGCCGCGAATTC
S_index3_right_PEPCR	CAAGCAGAAGACGGCATACGAGATGCCTAAGTGACTGGAGTTCAGACGTGTGCTCTTCCGATCTATGTGCAGGGCCGCGAATTC
S_index4_right_PEPCR	CAAGCAGAAGACGGCATACGAGATTGGTCAGTGACTGGAGTTCAGACGTGTGCTCTTCCGATCTATGTGCAGGGCCGCGAATTC
S_index5_right_PEPCR	CAAGCAGAAGACGGCATACGAGATCACTGTGTGACTGGAGTTCAGACGTGTGCTCTTCCGATCTATGTGCAGGGCCGCGAATTC
S_index6_right_PEPCR	CAAGCAGAAGACGGCATACGAGATATTGGCGTGACTGGAGTTCAGACGTGTGCTCTTCCGATCTATGTGCAGGGCCGCGAATTC
S_index7_right_PEPCR	CAAGCAGAAGACGGCATACGAGATGATCTGGTGACTGGAGTTCAGACGTGTGCTCTTCCGATCTATGTGCAGGGCCGCGAATTC
S_index8_right_PEPCR	CAAGCAGAAGACGGCATACGAGATTCAAGTGTGACTGGAGTTCAGACGTGTGCTCTTCCGATCTATGTGCAGGGCCGCGAATTC
Read1_seq_primer_GFP	GCTGGCACGCGTAAGAAGGAGATATAACCATG
**cloning primers**	
pCI_del_int_F (phospho)	GTGTCCACTCCCAGTTCAAT
pCI_del_int_R (phospho)	CTGCCCAGTGCCTCACGACC
mkate2_gibs_F	GATCCGCGTATGGTGGCCTTAAGATACATTGATGAG
mkate2_gibs_R	TGTAAGCGGATGCCGCACATGTTCTTTCCTGCG
pCI_gib_F	CGGCATCCGCTTACAGACAA
pCI_gib_R	CACCATACGCGGATCCTTATC
**qPCR primers**	
pcDNA5-UTR_F	GTTGCCAGCCATCTGTTGTT
pcDNA5-UTR_R	CTCAGACAATGCGATGCAATTTCC
pc5_5UTR_F	CCGGGACCGATCCAGCCTCC
pc5_3UTR_R1	GCAAACAACAGATGGCTGGC
pc5_3UTR_F	TAAGAATTCGCGGCCCTGC

pc5_INT_F	GAAGTTGGTCGTGAGGCACTG
pCI-UTR_F	CTTCCCTTTAGTGAGGGTTAATG
pCI-UTR_R	GTTTATTGCAGCTTATAATGGTTAC
pCI-mRNA_F	GCTAACGCAGTCAGTGCTTC
pCI-mRNA_R	ACACCCAGTGCCTCACGAC
pCI-premRNA_F	GAGGCACTGGGCAGGTAAGTATC
pCI-premRNA_R	GTGGATGTCAGTAAGACCAATAGGTG
Gapdh_F	GGAGTCAACGGATTTGG
Gapdh_R	GTAGTTGAGGTCAATGAAGGG
Neo_F	CCCGTGATATTGCTGAAGAG
Neo_R	CGTCAAGAAGGCGATAGAAG
LysCTT_F	TCAGTCGGTAGAGCATGAGAC
LysCTT_R	CAACGTGGGGCTCGAACC
Malat1_F	CAGACCCTTCACCCCTCAC
Malat1_R	TTATGGATCATGCCCACAAG
cMyc_F	CTCCTACGTTGCGGTCACAC
cMyc_R	CCGGGTCGCAGATGAAACTC
